# Dental‐implant inflamed surface area: A quantification and simulation study

**DOI:** 10.1002/JPER.24-0320

**Published:** 2025-03-24

**Authors:** Yumeng Yan, Praveen Sharma, Jeanie Suvan, Carlota Blanco, Antonio Linares, Yago Leira, Juan Blanco, Francesco D'Aiuto

**Affiliations:** ^1^ Periodontology Unit, Eastman Dental Institute University College London London UK; ^2^ Periodontal Research Group, School of Dentistry, Institute of Clinical Sciences University of Birmingham Birmingham UK; ^3^ National Institute for Health Research Birmingham Biomedical Research Centre Birmingham UK; ^4^ Birmingham Dental Hospital Birmingham Community Healthcare NHS Foundation Trust Birmingham UK; ^5^ Oral Sciences, University of Glasgow Dental School, School of Medicine, Dentistry and Nursing, College of Medical, Veterinary and Life Sciences University of Glasgow Glasgow UK; ^6^ Periodontology Unit, Faculty of Odontology, Health Research Institute of Santiago de Compostela University of Santiago de Compostela & Medical‐Surgical Dentistry Research Group Santiago de Compostela Spain

**Keywords:** dental implants, inflammation, peri‐implantitis

## Abstract

**Background:**

Peri‐implantitis is an oral inflammatory disease with increased incidence across the population as linked to the wide use of dental implants to replace missing teeth. While the recent World Workshop Classifications provided a framework for clinicians to diagnose and assess the severity of peri‐implantitis, an objective assessment of the mucosal inflammatory burden around the dental implant is still lacking. Based on the periodontal epithelium surface area (PESA) and periodontal inflamed surface area (PISA) scores previously reported, a study was conducted to explore a similar approach for peri‐implant inflammatory surface quantification. The aim of this study was to develop two novel scores of peri‐implant mucosal inflammation and their clinical application to help with the quantification of the dental‐implant inflamed surface area (DISA).

**Methods:**

Formulas were created to quantify the dental‐implant surface area (DESA), and then DESA of both tapered and cylinder implants was compared based on a dataset that included probing pocket depth, mucosal recession, and implant morphology parameters. The DISA was subsequently calculated using the epithelial/connective tissue areas multiplied by the proportion of bleeding on probing sites around the implant. The new scores were applied and validated using clinical cases of peri‐implantitis.

**Results:**

Firstly, a total of 10,000 dental implants were included in a simulation study to evaluate the performance of the new scores. Under‐estimation of the real surface areas around dental implants was less than 2% when using the DESA score for cylinder and tapered dental implants (universal formula is presented). The DISA score was created in the simulations and then applied to 21 participants suffering from peri‐implantitis. DESA scores ranged from 54.24 to 400.29 mm^2^, and the DISA scores ranged from 36.76 to 400.29 mm^2^.

**Conclusions:**

Two novel scores (DESA and DISA) to estimate the peri‐implant surface area in health and disease were proposed and applied to clinical cases. The inflammatory surface caused by peri‐implantitis could be quantified by DISA with good precision. Future steps could include microbiome assessments and investigation of the association of these scores with the host response and general health status of patients with peri‐implantitis.

**Plain Language Summary:**

Peri‐implantitis is a common gum disease surrounding dental implants. Although recent guidelines help diagnose it, there's still no clear way to measure the inflammation surface area around implants. We developed formulas to calculate the dental‐implant surface area (DESA) and compared them for different implant shapes using data on pocket depth, gum recession, and implant type. We then calculated the dental‐implant inflamed surface area (DISA) by multiplying the tissue area around the implant by the proportion of bleeding sites. In our study, we included data from 10,000 implants to test the new scores. The DESA score was very accurate, underestimating the real surface area by < 2%. We then used the DISA score on 21 patients with peri‐implantitis. The DESA scores ranged from 54.24 to 400.29 mm^2^, while the DISA scores ranged from 36.76 to 400.29 mm^2^. In conclusion, we introduced two new scores (DESA and DISA) to measure the inflammation caused by peri‐implantitis. These scores could help in better diagnosing and treating peri‐implantitis, with future research exploring their link to overall health.

## 1 INTRODUCTION

Periodontitis is a chronic inflammatory condition, initiated by bacteria, which, in susceptible individuals, leads to the destruction of the connective tissues and bone surrounding the teeth. Left untreated, periodontitis can result in tooth loss and systemic health complications. A similar disease process around dental implants is classified as peri‐implantitis.^1^

In both periodontitis and peri‐implantitis, bacteria or their end‐products and local inflammatory mediators are not only abundant at the gingival/mucosal level but they may spill over from inflamed tissue into the circulatory system, triggering a host response.^2^ The greater extent of gingival/mucosal tissue inflammation could represent an increased trigger for a systemic host response. To quantify the area of the periodontal tissue or wound, two measures of exposure have been identified as the periodontal epithelial surface area (PESA) and the periodontal inflamed surface area (PISA) by Nesse and co‐workers. Traditional clinical periodontal parameters including probing pocket depth (PPD), mucosal recession, and bleeding on probing (BOP), are used to calculate the PISA score.^3^ Recent evidence confirmed that the PISA values better reflect the systemic exposure of periodontal inflammation when compared with other measures of periodontitis, such as case definitions or mean PPD especially when assessing the link with systemic inflammation as assessed by C‐reactive protein (CRP) levels^4^, ^5^, ^6^ and kidney function.^7^

Similarly, peri‐implantitis has also been showing to be associated with several systemic diseases, such as cardiovascular disease,^8^, ^9^, ^10^ dyslipidemia,^11^ obesity,^12^ and diabetes.^13^ Compared with periodontitis, peri‐implantitis occurs more rapidly and it is characterized by a non‐linear and accelerating progression.^1^, ^14^ The mucosal inflammatory infiltrate is almost three times bigger when compared with that observed in the periodontitis lesion.^15^

The current assessment of peri‐implantitis severity/extent and its potential systemic impact is based only on PPD and bone loss evaluations,^1^ while an implant equivalent of PESA/PISA is yet to be proposed. A number of challenges can be identified when creating similar scores around dental implants. In particular, a dental implant's surface area depends on its diameter, length, and surface morphologies. Further, the inflamed surface area could be greater than that observed around natural teeth and clinicians might be using new indexes to compare different forms of peri‐implant diseases as well as response to existing or novel treatments. The aim of this study was to perform first a simulation and quantify the extent of underestimation in DESA/DISA in approximating a tapered implant as compared to a simple cylinder. We then propose two new scores (the dental‐implant surface area or DESA and the dental‐implant inflamed surface area or DISA) and their calculation and validation using commonly recorded parameters including implant diameter, PPD, mucosal recession, and BOP.

## 2 MATERIALS AND METHODS

### 2.1 Dental‐implant surface area (DESA)

Dental implants exist in three common macro‐morphologies (cylindrical, tapered, or a combination of both, that is, partly tapered). According to a comprehensive resource*, 1237 types of cylindrical implants and 1155 types of tapered dental implants were identified. In addition, the majority of tapered dental implants on the market are only narrowed/tapered when looking at the diameter of the apical third^16^ rather than the coronal‐apical portion. This study set out to calculate the hypothetical mucosal surface area around a dental implant based on the clinical PPD assessment as well as the evaluation of BOP.

#### 2.1.1 Calculating the surface area for cylinder implants

**FIGURE 1:**
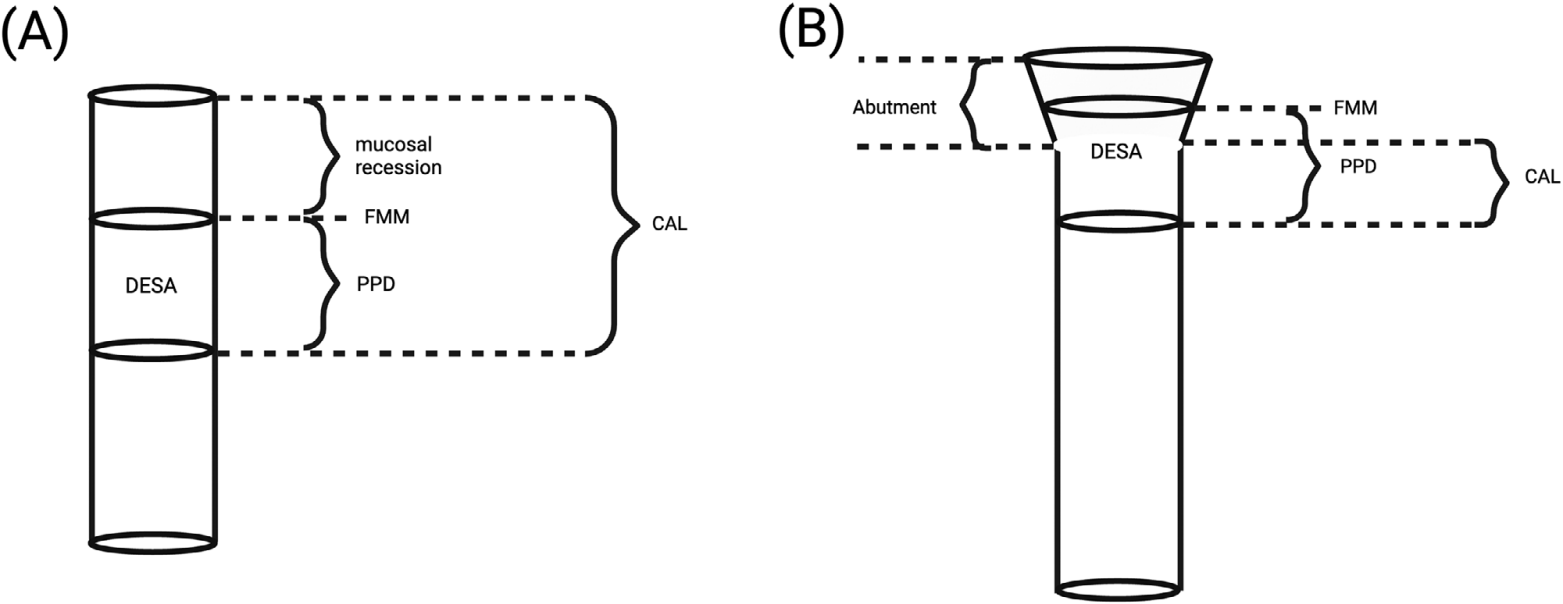
Dental‐implant surface area (DESA) of cylinder dental implants. (A) The free mucosal margin (FMM) is located below or at the level of the most coronal site of a dental implant, DESA refers to the surface around the dental implant. (B) The FMM is located above the most coronal part of the implant, indicating no mucosal recession in this scenario. In all these situations, the height of DESA corresponds to probing pocket depth (PPD). CAL, clincal attachment loss.

The diameter of a dental implant was taken as supplied by the manufacturer. The height of the cylinder in calculating the DESA was approximated as the mean PPD around that dental implant. It was envisaged two different scenarios would occur based on the location of the free mucosal margin (FMM):

1. If the FMM was below or at the level of the most coronal site of a dental implant, the DESA was referring to the surface around the dental implant (Figure 1A).†
2. If the FMM was above the level of the most coronal site of a dental implant, the DESA was referring to both the surface around the dental implant and the abutment. The diameter of the abutments was approximated as being the same as the dental implants in the formula for DESA. In reality, this would result in an underestimation of the DESA due to the diameter of abutments usually being greater than those of dental implants (Figure 1B).

The formula for the surface area calculation of cylinder implants (S_1_) is present in Table 1.

**TABLE 1:** Dental‐implant surface area (DESA) and dental‐implant inflamed surface area (DISA) calculation formulas and symbols.

Symbol	Definition	Description
*S_1_*	*π* * *d* * PPD	Dental‐implant surface area calculation of cylinder implants
*S_21_*	*π* * *d* * PPD	Dental‐implant surface area calculation of truncated cone (peri‐implant soft tissue was within the cylindrical part of the dental implant)
*S_22_*	*π* * *d* * (CPI‐MR) + *π* * (*R*_1_ + *R*_2_) * (mPPD/cos θ)	Dental‐implant surface area calculation of truncated cone (peri‐implant soft tissue spanned both the cylindrical and tapered part of the dental implant)
*S_23_*	*π* * (mPPD/cos θ) * (*R*_1_ + *R*_2_)	Dental‐implant surface area calculation of truncated cone (peri‐implant soft tissue lied entirely in the tapered part of the dental implant)
DISA	1/6 * (∑BOP) * DESA	Dental‐implant inflamed surface area

*Note*: *π*, 3.14159; *d*, diameter of the implant; *R*_1_, the implant radii of the coronal site of the peri‐implant pocket; *R*_2_, the implant radii of the apical site of the peri‐implant pocket; θ, angle of the tapered part; ∑BOP, cumulative for sites with bleeding on probing around each implant; mPPD, mean probing pocket depth around the dental implant; CPI, length of the cylindrical part of the dental implant; DESA, dental‐implant surface area; MR, mucosal recession.

#### 2.1.2 Calculating the surface area for tapered/partly tapered implants

**FIGURE 2:**
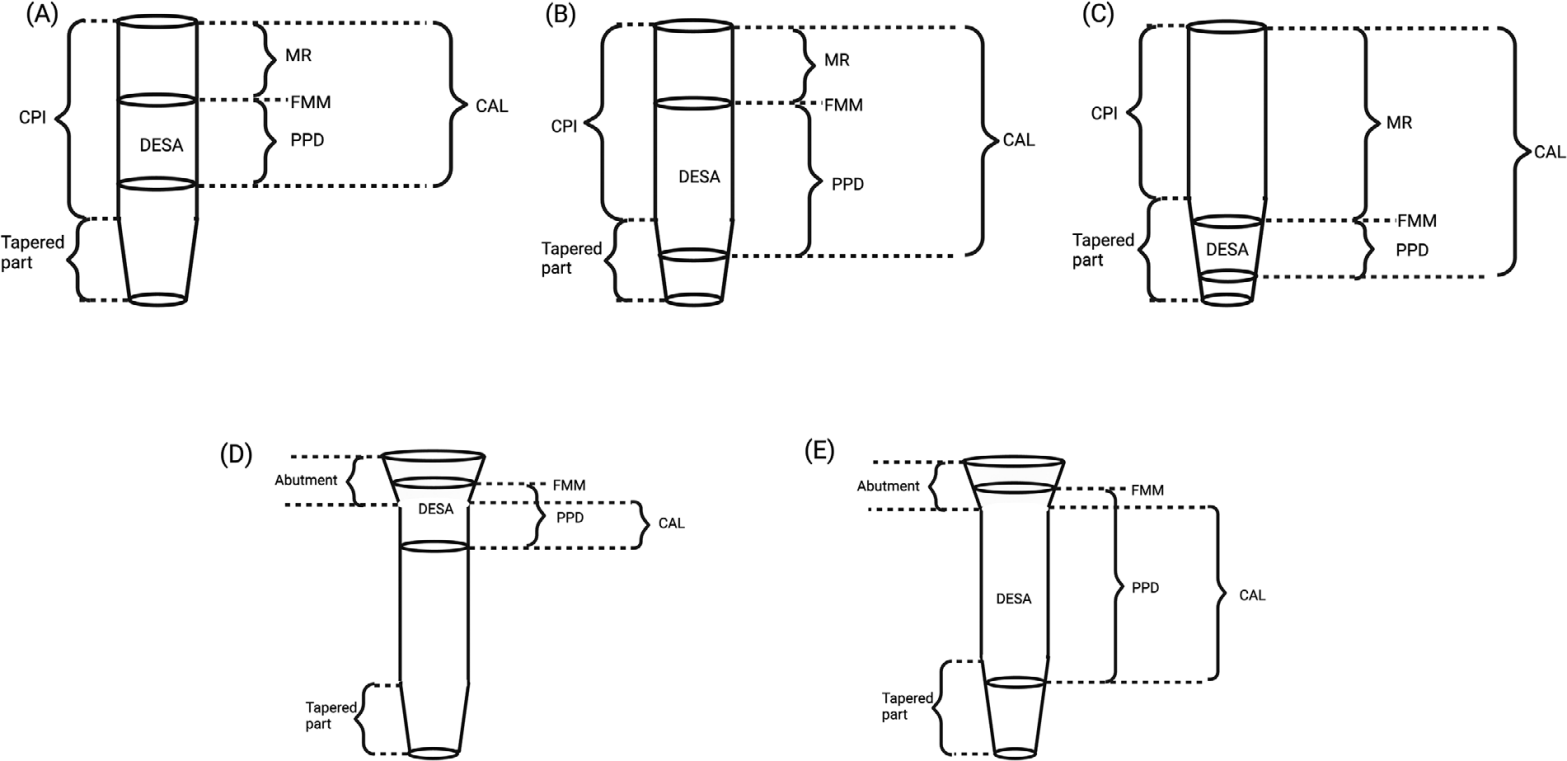
Dental‐implant surface area (DESA) of tapered dental implants. (A) DESA surrounds only the cylindrical part. The free mucosal margin (FMM) is below the most coronal site of the implant, clinical attachment loss (CAL) equals mucosal recession (MR) plus probing pocket depth (PPD), and the bottom of PPD is above the tapered portion. (B) The peri‐implant soft tissue spans both over the cylindrical and tapered part of the implant, DESA is the sum of the area of the cylindrical and truncated cone parts. (C) The peri‐implant soft tissue lies entirely within the tapered part of the implant, and DESA surrounds only the truncated cone. (D) The FMM is above the level of the most coronal site of implants, peri‐implant soft tissue spans both the cylindrical part and abutment. (E) The FMM is above the level of the most coronal site of implants, the peri‐implant soft tissue lies from the abutment to the tapered part. CPI, cylindrical part of the dental implant.

Using the formula of surface area calculation of truncated cone, the following situations were listed:

1. If the peri‐implant soft tissue, as measured by PPD, was within the cylindrical part of the dental implant (CPI) (Figure 2A)‡, the calculation for DESA (S_21_) was essentially the same as the cylinder above (Table 1).
2. If the peri‐implant soft tissue spanned both the cylindrical and tapered part of the dental implant then the calculation for the DESA (S_22_) was the sum of the area of the cylindrical and truncated cone part (Table 1) (Figure 2B).
3. If the peri‐implant soft tissue lied entirely in the tapered part of the dental implant the calculation for the DESA (S_23_) was based on that of a truncated cone (Table 1) (Figure 2C).
4. As with cylindrical implants, If the FMM was above the level of the most coronal site of the dental implant, the surface area was calculated using the PPD and the dental implant diameter as an approximation (Figure 2D,E).

### 2.2 Dental‐implant inflamed surface area (DISA)

To approximate the DISA, we used the methodology suggested by Nesse and co‐workers^3^ using BOP as a marker of mucosa inflammation. Hence, DISA was calculated as a product of DESA (Table 1).

Using these formulas, the calculations of DESA/DISA differ for cylindrical and tapered/partly tapered implants due to variations in taper degree and position. To create a universal formula that applies to all types of implants, a comparison of surface areas between cylindrical and tapered/partly tapered implants was performed.

#### 2.2.1 Details of the simulation

To compare the surface areas, firstly, a simulation dataset of 10,000 PPDs was created using a skewed‐t distribution with a minimum of 1 mm, a maximum of 15 mm, and a mode of 5 mm (see Figure S1A in the online *Journal of Periodontology*), This implies that the simulation uses a minimum PPD of 1 mm, a maximum of 15 mm, with 5 mm being the most frequently occurring PPD value in this distribution. Similarly, a dataset of 10,000 mucosal recession readings was created with a minimum of 0 mm, maximum of 10 mm, and mode of 2 mm (see Figure S1B in the online *Journal of Periodontology*). Dental implants of varying diameters (3.3, 4.1, and 4.8 mm), lengths (10, 12, and 14 mm) tapers (2°, 9°, and 14°) were modeled. Data were dropped if PPD + mucosal recession was greater than the length of the dental implant. All analyses were performed using statistical software.§

#### 2.2.2 DESA differences of cylinder and tapered implants

After excluding data where the sum of PPD and mucosal recession were greater than the length of the implant, the relative underestimation of the DESA score was compared between different morphologies after calculation of the surface area of the dental implants.

### 2.3 Clinical application

#### 2.3.1 Study population

Clinical and radiographic assessment of peri‐implantitis was provided from a previous study by Y.L.^11^ A total of 21 participants were included. Eligible participants diagnosed with peri‐implantitis were identified from the School of Dentistry at the University of Santiago de Compostela between January 2018 and 2019. The definition of peri‐implantitis was based on the 2018 Consensus Classification.^17^ Individuals diagnosed with at least one implant with peri‐implantitis were included.

#### 2.3.2 Clinical and radiographic examination

Clinical peri‐implant examinations were conducted on all participants by trained and calibrated periodontists. Calibration was completed before the study using 10 non‐study patients with peri‐implantitis. Intra‐examiner and inter‐examiner reliability were evaluated using the intraclass correlation coefficient (ICC) for PPD. The intra‐examiner ICC values were 0.88 and 0.85 for two independent examiners. The inter‐examiner ICC value was 0.84, indicating satisfactory measurement reliability.

Six sites (mesio‐buccal, disto‐buccal, mid‐buccal, mesio‐lingual/mesio‐palatal, disto‐lingual/disto‐palatal, and mid‐lingual/mid‐palatal) were measured for both PPD and BOP for all implants present, using a calibrated periodontal probe.‖

Each implant was assessed using an intraoral radiograph to evaluate crestal bone level and implant diameter (in millimeters). Digital periapical radiographs were obtained and reviewed on a calibrated computer screen.¶ Implant diameter was defined as the measurement of the neck site of the dental implant. Marginal bone loss was defined as the distance from the widest supracrestal part of the implant to the alveolar crest.

To examine the relationship between the DISA score and the number of inflamed dental implants, a Spearman correlation test was conducted. Data on DISA scores and the number of inflamed dental implants were collected for all participants, and the Spearman correlation coefficient (*ρ*) and significance level (*p*‐value) were calculated. The definition mentioned that probing depths ≥6 mm in conjunction with bleeding represents peri‐implantitis.^17^ To further validate the effectiveness of the DISA scores, participants were categorized into two classes based on the number of sites with PPD exceeding 6 mm: those with fewer than six sites of PPD ≥6 mm were classified as class one, while those with more than six sites of PPD ≥6 mm were classified as class two. Additionally, to classify inflammation burden using the DISA scores, an implant with a diameter of 4.1 mm and six PPD sites of 6 mm with BOP was used as a reference, corresponding to a DISA score of 77.28 mm^2^, which served as the classification threshold. Participants with a DISA score greater than 77.28 mm^2^ were categorized as having a moderate inflammation burden, while those with a DISA score below this threshold were categorized as having a mild inflammation burden. A confusion matrix was then constructed to evaluate the above classifications.

## 3 RESULTS

### 3.1 Calculation of the DESA

The highest underestimation among the tapered morphologies was 1.09% ± 2.17% with the length = 10 mm, taper = 14°, and diameter = 3.3 mm (see Figure S2A, Table S1 in online *Journal of Periodontology*). Dental implants with larger diameters but the same taper and length were associated with a relative underestimate of 0.82% ± 1.67% and 0.65% ± 1.36%, respectively (see Figure S2B,C, Table S1 in online *Journal of Periodontology*). The lowest underestimation among the tapered dental implants was 0.06% ± 0.20%, specifically linked to length = 14 mm, taper = 2°, and diameter = 4.8 mm. Dental implants with longer lengths, a larger diameter, and a smaller taper degree presented with lower relative underestimates (see Table S1 in the online *Journal of Periodontology*).

Based on the observed minimal underestimation, our calculations of the DESA scores were made by approximating all dental implant morphologies as simple cylinders.

This is the formula for the DESA (S) used:

S=π∗d∗mPPD

where *d* is the diameter of the neck site of dental implants and mPPD is the mean probing depth around the dental implant.

### 3.2 Calculation of the DISA

This formula for the DISA will combine DESA and BOP was used:

$$\mathrm{DISA}=1/6*\left(\Sigma \mathrm{BOP}\right)*\mathrm{DESA}$$

where BOP is the bleeding on probing around the implant and ∑BOP is the cumulative for sites with BOP.

A table was created using PPD, dental implant shape parameters, and BOP to calculate the DESA and DISA scores based on the above formulas (see Table S2 in the online *Journal of Periodontology*).

### 3.3 Validation of DESA/DISA

A total of 21 patients (mean age: 59.1 ± 10.0 years; 8 men) with 34 implants diagnosed with peri‐implantitis were included in the calculation of DESA/DISA scores. Implant‐based mean and standard deviation of PPD was 6.42 ± 1.51 mm, percentage of BOP was 91.18% ± 13.75%. Among these patients, 14 had a single dental implant with DESA scores ranging from 54.24 to 149.75 mm^2^ and DISA scores ranging from 36.75 to 149.75 mm^2^. Twelve of these patients had DESA/DISA scores both below 100 mm^2^, while only two had DESA/DISA scores >100 mm^2^, which was attributed to having either a wide implant of 5.6 mm or a mean PPD >10 mm.

Four patients had two dental implants, with DESA scores ranging from 116.24 to 154.67 mm^2^ and DISA scores ranging from 83.95 to 154.67 mm^2^. One patient had three dental implants, resulting in both a DESA and DISA score of 335.21 mm^2^, and one patient had four dental implants, with both DESA and DISA scores of 400.29 mm^2^. Another patient, with five dental implants, had a DESA score of 394.48 mm^2^ and a DISA score of 352.78 mm^2^.

The DESA scores ranged from 54.24 to 400.29 mm^2^, and the DISA scores ranged from 36.76 to 400.29 mm^2^ (Table 2). Then, a Spearman correlation test between the DISA score and the number of inflamed dental implants was performed. The result showed a Spearman correlation coefficient of 𝜌 = 0.7640 with a *p*‐value of 0.0001. The confusion matrix of these two classifications showed a sensitivity of 84.6%, specificity of 1, and accuracy of 90.5%. These results suggest that a larger area of mucosal inflammation is associated with higher DISA scores.

**TABLE 2:** Number, position of implants, DESA, and DISA scores for patients.

Patient ID	No. of implants	Position of implants	Diameter of implants (mm)	DESA (mm^2^)	DISA (mm^2^)
1	1	15	3.7	65.87	65.87
2	1	32	3.3	62.20	62.20
3	1	32	3.7	54.24	54.24
4	1	24	3.9	55.13	36.76
5	2	14, 25	4, 3.4	154.67	154.67
6	1	47	4	64.93	43.28
7	1	45	4.5	91.89	91.89
8	1	11	5.6	140.74	140.74
9	1	11	3.8	73.62	73.62
10	4	16, 14, 25, 26	3.7, 3.7, 4.3, 4.3	400.29	400.29
11	2	15, 24	3.7, 3.7	125.93	83.95
12	1	16	4.4	73.72	61.44
13	1	45	4.4	149.75	149.75
14	1	31	4.3	63.04	63.04
15	1	46	4.8	70.37	58.64
16	1	42	3.8	67.65	45.10
17	3	17, 16, 26	4.7, 4.5, 4.5	335.21	335.21
18	2	25, 23	3.7, 3.7	116.24	105.26
19	2	14, 33	4.7, 3.7	157.45	157.45
20	1	44	3.8	77.60	51.73
21	5	17, 16, 31, 35, 36	3.6, 4.1, 3.9, 3.9, 3.9	394.48	352.78

Abbreviations: DESA, dental‐implant surface areas; DISA, dental‐implant inflamed surface area.

## 4 DISCUSSION

In this study, two new scores for peri‐implant surface area were proposed as a proxy of local inflammatory burden applied to cases of peri‐implant disease. Approximation of all dental implant morphologies as simple cylinders leads to minimal underestimation when calculating the new scores DESA and DISA. Using commonly collected clinical parameters, such as the dental implant diameter, PPD and BOP, DESA and DISA scores could be easily calculated and provide insight into the level of peri‐implant tissue loss (see Table S2 in the online *Journal of Periodontology*).

This is the first attempt to create two novel dental implant mucosal channel scores to estimate the soft tissue inflammatory burden and also provide a tool for the assessment of systemic implications of peri‐implant diseases.

These new scores were created based on the large variety of lengths and diameters in dental implants reported. Lengths between 6 and 20 mm, with the most common being between 8 and 15 mm, and diameters on average from 3 to 7 mm,^18^ and the most common use of 4 mm^19^ were identified in this study. The findings suggest that the creation of new indexes around dental implants with longer lengths, larger diameters, and smaller degrees of taper tends to result in small underestimation.

This study revealed that higher levels of DISA scores represent higher mucosal inflammation. Both peri‐implant mucositis and peri‐implantitis are biofilm‐associated pathological conditions. The calculation of DESA and DISA relies on the mucosal channel around a dental implant, which is assessed by PPDs. Clinical assessment of the mucosal pocket can be difficult and open to subjective bias especially based on the thickness of the channel and the presence of different prosthetic/implant components; this is not readily compensated by just using radiographs. As with the PISA and PESA scores, no current thresholds of health/disease exist other than relative/changes, that is, a higher PISA/DISA score is worse. Future clinical studies will be needed to further characterize and better define the thresholds of DISA/DESA in health and disease.

The root surface area for different teeth ranges from 172 mm^2^ (lateral incisor) to 504 mm^2^ (first molar).^20^ When calculating around dental implants, the surface area is influenced by the diameter and length of the dental implant rather than its placement location. The diameters and lengths of dental implants are first assessed by reviewing previous dental implant placement medical records. If unavailable, the diameters are measured at the neck of the dental implants with digital periapical radiographs and corresponding calibrated software. This results in a lower variation in dental implant surface area when compared with natural teeth, regardless of the placement of dental implants. For instance, two dental implants with the same diameter and length placed in positions 12 and 16 will have identical surface areas. In our study, a patient had an implant at position 16 with a DESA of 73.72 mm^2^ and DISA of 61.43 mm^2^. Under the same conditions of PPD and BOP, the PESA and PISA measured 213.26 and 177.7 mm^2^, respectively. This scenario showed significantly lower levels of DESA/DISA.

On the contrary, one patient had an implant at position 11 with a DESA of 73.62 mm^2^ and DISA of 73.62 mm^2^. Under the same conditions of PPD and BOP, the PESA and PISA were 112.4 mm^2^ each. Another patient also had an implant at position 11, with DESA/DISA measuring 140.74 mm^2^ each. With the same PPD and BOP, the PESA and PISA were similar to DESA/DISA at 144.58 mm^2^ each. PESA/PISA in these two cases changed from 112.4 to 144.58 mm^2^, while DESA/DISA changed from 73.62 to 140.74 mm^2^. This indicates that for the same tooth position, DESA/DISA can vary significantly due to the choice of implant diameter. The variation in DESA between different positions is less pronounced compared with PESA. This is mainly because DESA is influenced by the implant diameter and PPD, whereas PESA is influenced by the natural tooth surface. Particularly in molars, PESA always exceeds DESA under identical PPD conditions.

Currently, objective tools a clinician could use to quantify the local mucosal inflammatory response are non‐existent. The proposed new scores could be investigated further, especially when histological confirmation of inflammatory infiltrates in mucosal tissues is available to define the relationship of the inflammatory score and the size of the inflammatory infiltrate. Based on the current knowledge, it is recognized that patients with peri‐implantitis can exhibit similar symptoms to those suffering from periodontitis.^21^ DISA is an estimate of the mucosal inflamed surface area at the implant level or patient level in the case of multiple implants. It is a mere estimate of a surface extension to the same extent that the PISA score provides around teeth. In cases when a patient presents with both peri‐implantitis/peri‐implant mucositis and periodontitis, we would suggest considering a combination of DESA/DISA and PESA/PISA scores as to calculate the total surface. This is, however, still an underestimation of the histopathological, microbiological, and immunological characteristics of the gingival and mucosal tissues. Akin to PESA, DESA relies on the PPD hence would be greater for implants placed deeper due to the corresponding increase in probing depth. As with PISA, the inflammatory component of DISA relies on the presence of BOP as a surrogate for local inflammation. The DISA score would not be providing information about the underlying cause of BOP. The assumption of our calculations is that the clinician would be competent enough to define whether BOP around a dental implant would be a sign of mucosal inflammation. Further, it would always be dependent upon the clinical judgment of the assessing operator as to whether certain BOPs should be included in the calculation of DISA.

While this is the first proposition of a new inflammatory score for assessing peri‐implant mucosal inflammation, there are some limitations to be considered. It is important to highlight to the reader that our simulations refer merely to the mucosal surface area rather than being a true reflection of the tissue inflammatory infiltrate/content. The accuracy of the DESA score is influenced by several factors. Firstly, in the calculations, a dental implant was considered to be a cylinder without threads. The microstructure, thread depth, width, and pitch could affect the DESA accuracy, resulting in an underestimation of the real surface area. However, while these features would increase the surface area of intimate contact with the dental implant (such as bone), they are likely to have a lesser impact on any soft tissue in contact with the dental implant when assessed by PPD. Indeed, digital measurement of the DESA is more suitable for obtaining accurate scores. Cone‐beam computed tomography can be used to measure the diameters and lengths of dental implants. However, measuring the soft tissue around dental implants with cone‐beam computed tomography remains challenging. Additionally, not all patients with peri‐implant diseases undergo cone‐beam computed tomography for each treatment due to the severity of the diseases, radiation exposure, and cost considerations. This may require the development of new equipment that does not currently exist. Future studies should focus on exploring this approach. Secondly, if the peri‐implant mucosa is overgrown (i.e., the FMM extends beyond the dental implant), the DESA score could also underestimate the surface area because the abutment diameter is greater than the implant diameter (Figure 2D,E). A retrospective study, however, demonstrated that peri‐implantitis is almost always accompanied by mucosal recession hence overgrowth is relatively infrequent.^22^ Then as most of the tapered dental implants manufactured are narrowed only in the apical third, in this study, we considered the difference between a cylindrical or tapered dental implant to be minimal. Another challenge is the potential operator‐dependent errors. Accurately measuring PPD around dental implants can be difficult due to the complexity of the implant surface and the shape of the crowns. The accuracy of BOP also rely upon the clinician as it might be due more to probing pressure rather than actual inflammation. All clinical scores/parameters may not provide accurate predictions of the mucosal inflamed area as prone to measurement bias. Although several bleeding scales exist, such as the Implant Mucosal Index, BOP remains the most widely used method. Additionally, BOP can be used to determine the percentage of bleeding around dental implants, making it particularly useful for calculating the DISA scores. Furthermore, the accuracy of the scoring systems may also be influenced by the limitation of measuring only two dimensions. The infiltrated tissues extend beyond the surface, their thickness may also contribute to the overall inflammatory burden. Future research should plan to apply these scores to larger cohorts of patients with peri‐implant mucositis and varying degrees of peri‐implantitis. In particular recruiting participants with multiple sites affected by peri‐implant inflammation would allow better comparison/quantification of the inflammatory burden. Incorporating measures of mucosal thickness as well as inflammatory markers should enhance the comprehensiveness of the assessments.

The strengths of this study include the robust methodology, varying dental implant diameters, lengths, and tapers used to ensure that the worst‐case scenario in the underestimation of DESA from approximating tapered implants as cylinders was clinically acceptable. In addition, strict clinical validation with a group of peri‐implantitis patients was conducted with the formulas.

## 5 CONCLUSIONS

In conclusion, two novel scores (DESA and DISA) to estimate the peri‐implant inflamed surface area in health and disease were proposed. The modeling and testing performed confirmed little under‐estimation of the real surface areas. The clinical application indicates that inflammatory surface caused by peri‐implantitis could be quantified by DISA with reasonable precision. Further steps should be taken in investigating both in observational and experimental studies the relationship between DISA and DESA with the local mucosal host‐response (i.e., inflammation) as well as the microbial profile at the peri‐implant lesion.

## AUTHOR CONTRIBUTIONS

Yumeng Yan, Praveen Sharma, and Francesco D'Aiuto have contributed substantially to the conception and design of the study and conceived the initial idea for this article. Yumeng Yan and Praveen Sharma developed the formulas and conducted the simulations. Carlota Blanco, Antonio Linares, Yago Leira, and Juan Blanco provided clinical data and interpretation of analyses. Francesco D'Aiuto critically evaluated these formulas. Yumeng Yan, Praveen Sharma, and Francesco D'Aiuto were involved in the interpretation of the data and drafted the manuscript. All the authors revised the manuscript critically. All authors have given final approval of the version to be published.

## CONFLICT OF INTEREST STATEMENT

The authors declare no conflicts of interest.

## PATIENT CONSENT FOR PUBLICATION

Yes.

## Supporting information



Supporting information

Supporting information

Supporting information

Supporting information

## Data Availability

All data relevant to the study are available on request form the corresponding author for a period or 24 months from the date of publication.

## Footnotes

* osseosource.com.

† Created in BioRender. Yan, Y. (2023) BioRender.com/f10g346.

‡ Created in BioRender. Yan, Y. (2023) BioRender.com/j69l859.

§ Stata 18.0 MP‐Parallel Edition, StataCorp, College Station, Texas, USA.

‖ 15 UNC Color‐Coded Probe, Hu‐Friedy, Chicago, Illinois, USA.

¶ Digora, KaVo Dental, Biberach an der Riss, Germany.
