# Supplementary material for: Dental‐implant inflamed surface area: A quantification and simulation study
Source: J Periodontol. 2025 Mar 24;96(9):994–1003. doi: 10.1002/JPER.24-0320 (PMC12447368; doi:10.1002/JPER.24-0320)
Supplement: Supplementary file 1 — Supporting information [file JPER-96-994-s002.docx]

Supplementary Table.1 Underestimates of dental-implant surface area (DESA) between cylinder and tapered dental implants.

| Taper and diameter of dental implant | Lenth of dental implant | | |
| --- | --- | --- | --- |
|  | Length 10mm | Length 12mm | Length 14mm |
| taper = 2°,  diameter =3.3mm | 0.19% ±0.35% | 0.14%±0.35% | 0.08%±0.30% |
| taper = 9°,  diameter =3.3mm | 0.75%±1.45% | 0.58%±1.49% | 0.36%±1.28% |
| taper = 14°,  diameter = 3.3mm | 1.09%±2.17% | 0.86%±2.26% | 0.53%±1.95% |
| taper = 2°, diameter = 4.1mm | 0.15%±0.28% | 0.11%±0.28% | 0.07%±0.24% |
| taper = 9°, diameter = 4.1mm | 0.58%±1.13% | 0.45%±1.17% | 0.28%±1.01% |
| taper = 14°, diameter = 4.1mm | 0.82%±1.67% | 0.65%± 1.74% | 0.40%±1.52% |
| taper = 2°, diameter = 4.8mm | 0.13%±0.24% | 0.09%±0.23% | 0.06%±0.20% |
| taper = 9°, diameter = 4.8mm | 0.48%±0.94% | 0.37%±0.98% | 0.23%±0.84% |
| taper = 14°, diameter = 4.8mm | 0.65%±1.36% | 0.53%±1.43% | 0.33%±1.25% |
