# Supplementary material for: Dental‐implant inflamed surface area: A quantification and simulation study
Source: J Periodontol. 2025 Mar 24;96(9):994–1003. doi: 10.1002/JPER.24-0320 (PMC12447368; doi:10.1002/JPER.24-0320)
Supplement: Supplementary file 2 — Supporting information [file JPER-96-994-s004.docx]

Supplementary Table 2. DESA and DISA calculation ^[[1]](#footnote-1)^

*DESA(mm^2^)= π * d * meanPPD*

*DISA(mm^2^)= 1/6 * (∑BoP) * DESA*

d=diameter of the dental implant

PPD=probing pocket depth

BoP=1 if bleeding exist, BoP=0 if no bleeding

MB=mesio–buccal

B=mid-buccal

DB=disto–buccal

ML/MP=mesio–lingual/palatal

L/P=mid-lingual/palatal

DL/DP=disto–lingual/palatal

meanPPD=mean probing pocket depth around the dental implant

∑BoP= cumulative for sites with BoP

1. Double click the above table or Excel icon to open an Excel spreadsheet for the calculation of DESA/DISA [↑](#footnote-ref-1)
