# Supplementary material for: Dental‐implant inflamed surface area: A quantification and simulation study
Source: J Periodontol. 2025 Mar 24;96(9):994–1003. doi: 10.1002/JPER.24-0320 (PMC12447368; doi:10.1002/JPER.24-0320)
Supplement: Supplementary file 3 — Supporting information [file JPER-96-994-s001.docx]

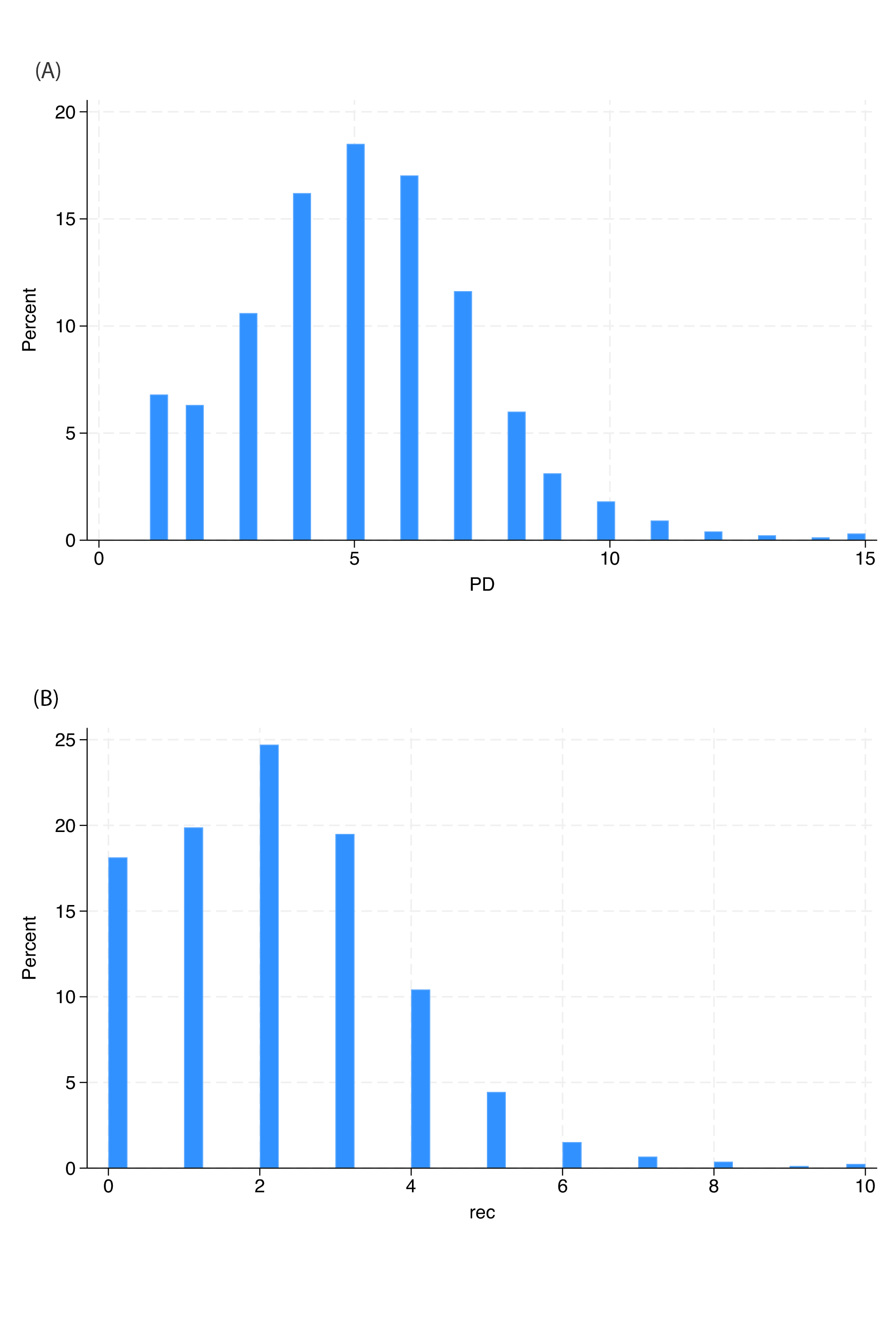


Supplementary FIGURE.1 Skewed distribution of probing pocket depth (PPD) and mucosal recession.

(A). Skewed distribution of probing pocket depth (PPD). X axis, PD (mm): PPD. Y axis, percent: percentage of PPD. PPD ranges from a minimum of 1 mm to a maximum of 15 mm, with a mode of 5 mm. (B). Skewed distribution of mucosal recession. X axis, rec(mm): mucosal recession. Y axis, percent: percentage of mucosal recession. Mucosal recession ranges from a minimum of 0 mm to a maximum of 10 mm, with a mode of 2 mm.
