# Supplementary material for: Dental‐implant inflamed surface area: A quantification and simulation study
Source: J Periodontol. 2025 Mar 24;96(9):994–1003. doi: 10.1002/JPER.24-0320 (PMC12447368; doi:10.1002/JPER.24-0320)
Supplement: Supplementary file 4 — Supporting information [file JPER-96-994-s003.docx]

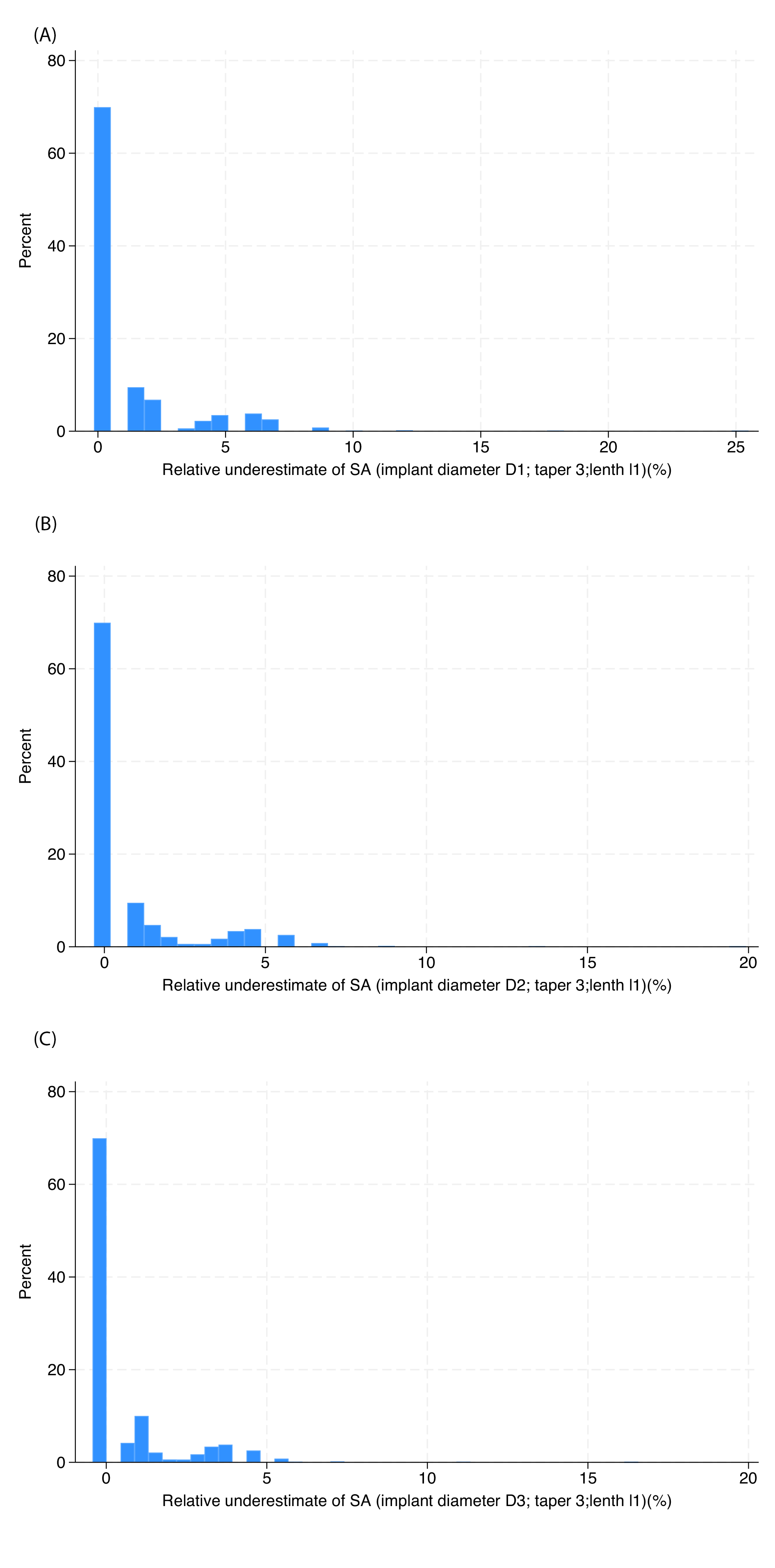


Supplementary FIGURE. 2 Relative underestimate of dental-implant surface area (DESA) of tapered and cylinder dental implants.

2A. X axis: relative underestimate of surface area with the dental implants of diameter 3.3mm, taper 14°, length 10mm. 2B. X axis: relative underestimate of surface area with the dental implants of diameter 4.1mm, taper 14°, length 10mm.2C. X axis: relative underestimate of surface area with the dental implants of diameter 4.8 mm, taper 14°, length 10mm.
